# The pro-fibrotic properties of transforming growth factor on human fibroblasts are counteracted by caffeic acid by inhibiting myofibroblast formation and collagen synthesis

**DOI:** 10.1007/s00441-015-2285-6

**Published:** 2015-10-09

**Authors:** Masum M. Mia, Ruud A. Bank

**Affiliations:** Department of Pathology & Medical Biology, University of Groningen, University Medical Center Groningen, Hanzeplein 1, 9713 GZ Groningen, The Netherlands

**Keywords:** Fibrosis, Collagen, Myofibroblasts, Caffeic acid, Lysyl hydroxylase

## Abstract

Fibrosis is a chronic disorder affecting many organs. A universal process in fibrosis is the formation of myofibroblasts and the subsequent collagen deposition by these cells. Transforming growth factor beta1 (TGFβ1) plays a major role in the formation of myofibroblasts, e.g. by activating fibroblasts. Currently, no treatments are available to circumvent fibrosis. Caffeic acid phenethyl ester (CAPE) shows a broad spectrum of biological activities, including anti-fibrotic properties in vivo in mice and rats. However, little is known about the direct effects of CAPE on fibroblasts. We have tested whether CAPE is able to suppress myofibroblast formation and collagen formation of human dermal and lung fibroblasts exposed to TGFβ1, and found that this was indeed the case. In fact, the formation of myofibroblasts by TGFβ1 and subsequent collagen formation was completely abolished by CAPE. The same was observed for fibronectin and tenascin C. The lack of myofibroblast formation is likely due to the suppression of *GLI1* and *GLI2* expression by CAPE because of diminished nuclear SMAD2/3 levels. Post-treatment with CAPE after myofibroblast formation even resulted in a partial reversal of myofibroblasts into fibroblasts and/or reduction in collagen formation. Major discrepancies were seen between mRNA levels of collagen type I and cells stained positive for collagen, underlining the need for protein data in fibrosis studies to make reliable conclusions.

## Introduction

Caffeic acid phenethyl ester (CAPE) is a polyphenolic compound with major anti-inflammatory, anti-fibrotic, anti-oxidant and immune-modulatory properties (Tolba et al. 2013; Murtaza et al. 2014). CAPE is one of the active compounds of honeybee propolis (Banskota et al. 2001), and has been tested in vivo in a variety of fibrosis-related models in rats and mice (Larki et al 2013; Tomur et al. 2011; Ogeturk et al. 2005; Özyurt et al. 2004; Zhao et al. 2014; Chuang et al. 2014, 2015). These studies showed a protective effect of CAPE, i.e. it attenuated the hallmark of fibrosis, namely excessive collagen deposition. Collagen is produced by myofibroblasts (Goldsmith et al. 2014; Mallat et al. 2013), and the in vivo studies indeed revealed a decrease in the amount of alpha-smooth muscle actin (αSMA)-positive cells, a marker that is widely used to detect myofibroblasts. However, little is known about the mechanisms behind the anti-fibrotic properties of CAPE. Fibrosis is an end result of the tissue repair process, and in this process a variety of inflammatory cells are involved, cells that secrete during the course of healing various cytokines that stimulate myofibroblast formation. Because of the many different inhibitory properties of CAPE shown in vivo and in vitro, animal studies cannot reveal whether CAPE directly acts on fibroblasts. Thus, it is not known if the decrease in myofibroblast numbers seen in vivo is an effect of the anti-inflammatory or immuno-modulatory properties of CAPE, or, alternatively, whether CAPE also directly affects the transformation of fibroblasts into myofibroblasts.

To the best of our knowledge, only some fragmentary information is available on the direct effect of CAPE on fibroblasts regarding expression/synthesis of collagen type I and αSMA. Murine lung fibroblasts (L929 cell line) exposed to bleomycin exhibited increased levels of collagen production (as measured by the collagen-specific amino acid hydroxyproline in the culture medium); this was not the case when the cells were first pretreated with CAPE (Liu et al. 2013). On the other hand, NIH 3T3 murine cells treated with CAPE showed an increase of intracellular collagen levels (Song et al. 2008). Hepatic stellate cells of Wistar rats exposed to CAPE showed a decreased collagen type I gene expression (Zhao et al. 2003). Finally, human nasal polyp-derived fibroblasts treated with TGFβ1 showed increased gene and protein levels of αSMA and collagen types I and III; this was inhibited by pretreating the fibroblasts with CAPE (Chun et al. 2014).

Transforming growth factor beta1 (TGFβ1) is one of the strongest pro-fibrotic cytokines (Ghosh et al. 2013; Yue et al. 2010). Fibroblasts stimulated with TGFβ1 rapidly transform into myofibroblasts and start to produce large amounts of collagen type I (Goldsmith et al. 2014; Mallat et al. 2013). In the current study, we have stimulated human adult dermal (HDFa) and human adult lung (HLFa) fibroblasts with TGFβ1 in the presence or absence of CAPE, and investigated the expression and/or synthesis of cytoskeletal components, collagen type I, the collagen-modifying enzyme lysyl hydroxylase 2 (LH2), and the transcription factors GLI1, GLI2, and SNAIL. In addition, we investigated whether myofibroblasts that are formed by TGFβ1 can be reversed into fibroblasts by a post-treatment with CAPE.

## Materials and methods

### Cell culture

HDFa [Caucasian, 20 years, CCD-1093Sk (ATCC^®^ CRL-2115™), ATCC, USA] and HLFa [Caucasian, 27 years, CCD-19Lu (ATCC^®^ CCL-210™), ATCC, USA] were cultured in basal medium [= Eagle’s minimal essential medium (BE12-662F; Lonza, Switzerland) containing 1 % l-glutamine (Lonza), 1 % penicillin/streptomycin (Gibco Life Technologies, UK)] supplemented with 10 % fetal bovine serum (FBS) (Thermo Scientific, USA). Passages 5–10 of HDFa and HLFa were seeded with a density of 15,000 cells/cm^2^ in a Costar 12-well plate (for quantitative real time polymerase chain reaction) or in a 48-well plate/8-well chamber slides (Corning, USA; for immunofluorescence staining). After 72 h, fibroblasts were washed with phosphate-buffered saline (PBS), starved overnight in basal medium supplemented with 0.5 % FBS, treated for 30 min in basal medium supplemented with 0.17 mM L-ascorbic acid 2-phosphate magnesium salt (A-8960; Sigma, USA) and 0.5 % FBS with/without CAPE (5 μg/ml) (2743; Tocris, UK), and subsequently cultured for 48 h in basal medium supplemented with 0.17 mM L-ascorbic acid 2-phosphate magnesium salt and 0.5 % FBS with/without recombinant human TGFβ1 (10 ng/ml) (100-21; Peprotech, UK) in the presence/absence of CAPE (5 μg/ml). In another experiment, the fibroblasts that were starved overnight were stimulated with basal medium supplemented with 0.17 mM L-ascorbic acid 2-phosphate magnesium salt and 0.5 % FBS with/without TGFβ1 (10 ng/ml) for 48 h, followed by a post-treatment with basal medium supplemented with 0.17 mM L-ascorbic acid 2-phosphate magnesium salt and 0.5 % FBS with/without CAPE (5 μg/ml) for 24 h. Subsequently, whole-cell lysates (as obtained with FARB Buffer; Favorgen Biotech, Taiwan) were used for quantitative real time polymerase chain reaction (qRT-PCR). For immunofluorescence studies, cells were washed with PBS and fixed with methanol/acetone (1:1 ratio; Merck, Germany) for 5 min. The CAPE compound was dissolved in absolute ethanol (vehicle) at a concentration of 25 μg/μl. All cell culture protocols were performed at 37 °C in a humidified 5 % CO_2_ environment.

### RNA isolation, cDNA synthesis and qRT-PCR

Total RNA was isolated using the Favorgen RNA extraction kit (Favorgen Biotech) according to the manufacturer’s protocol. The concentration and quality of RNA was measured with UV spectrophotometry (NanoDrop Technologies, Wilmington, NC, USA). For the synthesis of cDNA, total RNA was reverse transcribed with the First Strand cDNA synthesis kit (Fermentas, Lithuania) according to the manufacturer’s protocol. Gene expression analysis was performed by means of qRT-PCR in a 10-μl reaction mixture containing 10 ng cDNA, SYBR Green Master Mix (Roche, USA), 6 μM forward primer and 6 μM reverse primer (for primer sequences, see Table 1). qRT-PCR was conducted in triplicate for each condition in a 384-well plate at 95 °C for 15 s and 60 °C for 1 min for 40 cycles using the ViiA 7 Real-Time PCR System (Applied Biosystems, USA). Data were analyzed with the ViiA 7 Real-Time PCR System Software (Applied Biosystems). All mRNA data were normalized against the reference gene tyrosine 3-monooxygenase/tryptophan 5-monooxygenase activation protein, zeta isoform (YWHAZ).

**Table 1:** List of primer sequences used for qRT-PCR

Gene	Forward sequence	Reverse sequence
*ACTA2*	CTGTTCCAGCCATCCTTCAT	TCATGATGCTGTTGTAGGTGGT
*TAGLN*	GGCCAAGGCTCTACTGTCTG	CCCTTGTTGGCCATGTCT
*COL1A1*	GGGATTCCCTGGACCTAAAG	GGAACACCTCGCTCTCCA
*COL1A2*	CTGGAGAGGCTGGTACTGCT	AGCACCAAGAAGACCCTGAG
*PLOD2*	ATGGAAATGGACCCACCAA	TGCAGCCATTATCCTGTGTC
*GLI1*	CAGGGAGGAAAGCAGACTGA	ACTGCTGCAGGATGACTGG
*GLI2*	CACGCTCTCCATGATCTCTG	CCCCTCTCCTTAAGGTGCTC
*SNAIL1*	GCTGCAGGACTCTAATCCAGA	ATCTCCGGAGGTGGGATG
*FN1*	CTGGCCGAAAATACATTGTAAA	CCACAGTCGGGTCAGGAG
*TNC*	CCGGACCAAAACCATCAGT	GGGATTAATGTCGGAAATGGT
*YWHAZ*	GATCCCCAATGCTTCACAAG	TGCTTGTTGTGACTGATCGAC

### Immunofluorescence staining

After methanol/acetone fixation, fibroblasts were washed and incubated with primary antibodies (Table 2) diluted in PBS containing 2 % bovine serum albumin (BSA) (K1106; Sanquin, Netherlands) for 1 h at RT. After washing with PBS, cells were incubated for 30 min at RT with biotinylated secondary antibodies (Table 3) diluted in PBS containing 2 % BSA for 30 min at RT. The cells were washed again and incubated with streptavidin-CY3 (Invitrogen, USA) (1:100) in PBS containing 1 % BSA and DAPI (1:10,000) for 30 min. After washing with PBS, cell culture wells were mounted with Citifluor (Agar Scientific, UK) and the staining pattern was visualized with fluorescence imaging microscopy (TissueFAXS; TissueGnostics, Austria). TissueFAXS data was analyzed with the TissueQuest software as described previously (Mia et al. 2014).

**Table 2:** List of primary antibodies used for immunofluorescence analysis

Antigen (target protein)	Antibody (dilution)	Source (Cat. #, Company)
αSMA	Mouse monoclonal IgG2a (1:100)	M0851, Dako, Denmark
SM22α	Polyclonal rabbit IgG (1:200)	ab14106, Abcam, UK
Collagen type I	Mouse monoclonal IgG1 (1:300)	ab90395, Abcam, UK
LH2	Mouse polyclonal IgG (1:100)	SAB1400213, Sigma, USA
Fibronectin	Rabbit polyclonal IgG (1:400)	ab6584, Abcam, UK
Tenascin C	Mouse monoclonal IgG1 (1:100)	ab6393, Abcam, UK
SMAD2/3	Polyclonal goat IgG (15μg/ml)	AF3797, R&D, UK

**Table 3:** List of secondary antibodies used for immunofluorescence analysis

Antigen (target protein)	Biotinylated secondary antibody (dilution 1:100)	Source (Cat. #, Company)
αSMA	Goat-anti-mouse IgG2a	1080-08, SouthernBiotech, USA
SM22α and fibronectin	Goat anti-rabbit IgG	E0432, Dako, Denmark
Collagen type I and tenascin C	Goat-anti-mouse IgG1	1071-08, SouthernBiotech, USA
LH2	Goat-anti-mouse IgG	1030-08, SouthernBiotech, USA
Smad2/3	Rabbit-anti-goat IgG	6160-08, SouthernBiotech, USA

### Nuclear/cytoplasmic localization of SMAD2/3

HDFa and HLFa were cultured for 45 min in the presence of CAPE alone, TGFβ1 alone, or TGFβ1 in combination with CAPE. After treatment, cells were washed with ice-cold PBS and fixed with methanol/acetone (1:1) for 5 min. Subsequently, cells were washed with PBS and incubated with polyclonal goat anti-human to SMAD2/3 (AF3797; R&D, UK) diluted to 15 μg/ml in PBS containing 2 % bovine serum albumin (BSA) (K1106; Sanquin, Netherlands) for 3 h at 4 °C. After washing with PBS, cells were incubated with biotinylated secondary antibody rabbit anti-goat (6160-08; SouthernBiotech, USA) diluted in PBS containing 2 % BSA for 30 min at RT. The cells were washed again and incubated for 30 min with streptavidin-CY3 (Invitrogen) (1:100) in PBS containing 1 % BSA and DAPI (1:10,000). After washing with PBS, cell culture wells were mounted with Citifluor (Agar Scientific) and the staining pattern was visualized by using confocal laser scanning microscopy (Leica TCS SP8; Leica Microsystems, Germany).

### Statistics

All mRNA and immunofluorescence data are presented as mean ± SEM for at least three independent experiments. Results were analysed with either one-way analysis of variance (ANOVA) followed by Tukey’s post-test or two-tailed unpaired *t* test analysis using Graph-Pad Prism v.5 (GraphPad Software, USA). *P* < 0.05 was considered to be statistically significant.

## Results

### Basal levels of mRNA and protein in unstimulated HDFa and HLFa

**Fig. 1:**
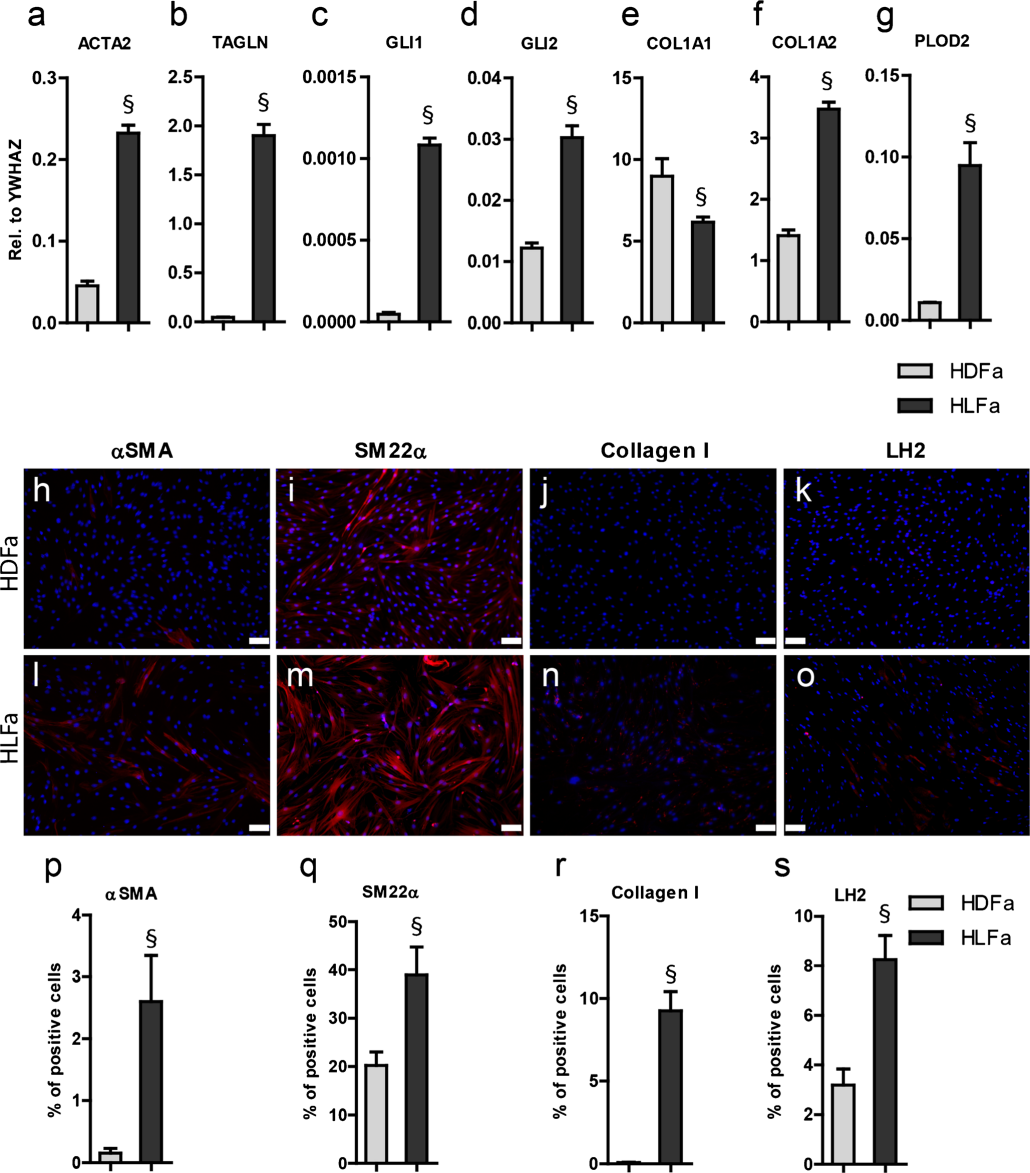
Characterization of mRNA levels and protein synthesis as observed in non-activated HDFa and HLFa. Fibroblasts were cultured for 48 h in basal medium supplemented with 0.17 mM L-ascorbic acid 2-phosphate magnesium salt and 0.5 % FBS in the absence of TGFβ1 and CAPE. **a**–**g** mRNA levels of *ACTA2*, *TAGLN*, *GLI1*, *GLI2*, *COL1A1*, *COL1A2* and *PLOD2* relative to the reference gene *YWHAZ*. **h**–**o** Representative immunofluorescence stainings (*upper panel*) and **p**–**s** quantification of the % of cells (*lower panel*) positive for αSMA, SM22α, collagen type I and LH2. § Statistically significant towards HDFa. *Scale bar* 100 μm

There were major differences in the basal expression levels between HDFa and HLFa (Fig. 1). In HLFa, a higher expression was seen of *ACTA2* (Fig. 1a), *TAGLN* (Fig. 1b), *GLI1* (Fig. 1c), *GLI2* (Fig. 1d), *COL1A2* (Fig. 1f), and *PLOD2* (Fig. 1g) compared to HDFa (a 4-fold, 5-fold, 10-fold, 2-fold, 2-fold and 4-fold difference, respectively). The basal expression of *COL1A1* was slightly, but significantly, lower in HLFa compared to HDFa (Fig. 1e). The major differences in basal expression were also reflected in the protein stainings for αSMA, SM22α, collagen I and LH2: the % cells that stained positive for these proteins was always higher in HLFa than HDFa (6-fold, 2-fold, 20-fold and 2.5-fold difference, respectively) (Fig. 1h–s).

### Effect of TGFβ1 and CAPE on nuclear localization of SMAD2/3

**Fig. 2:**
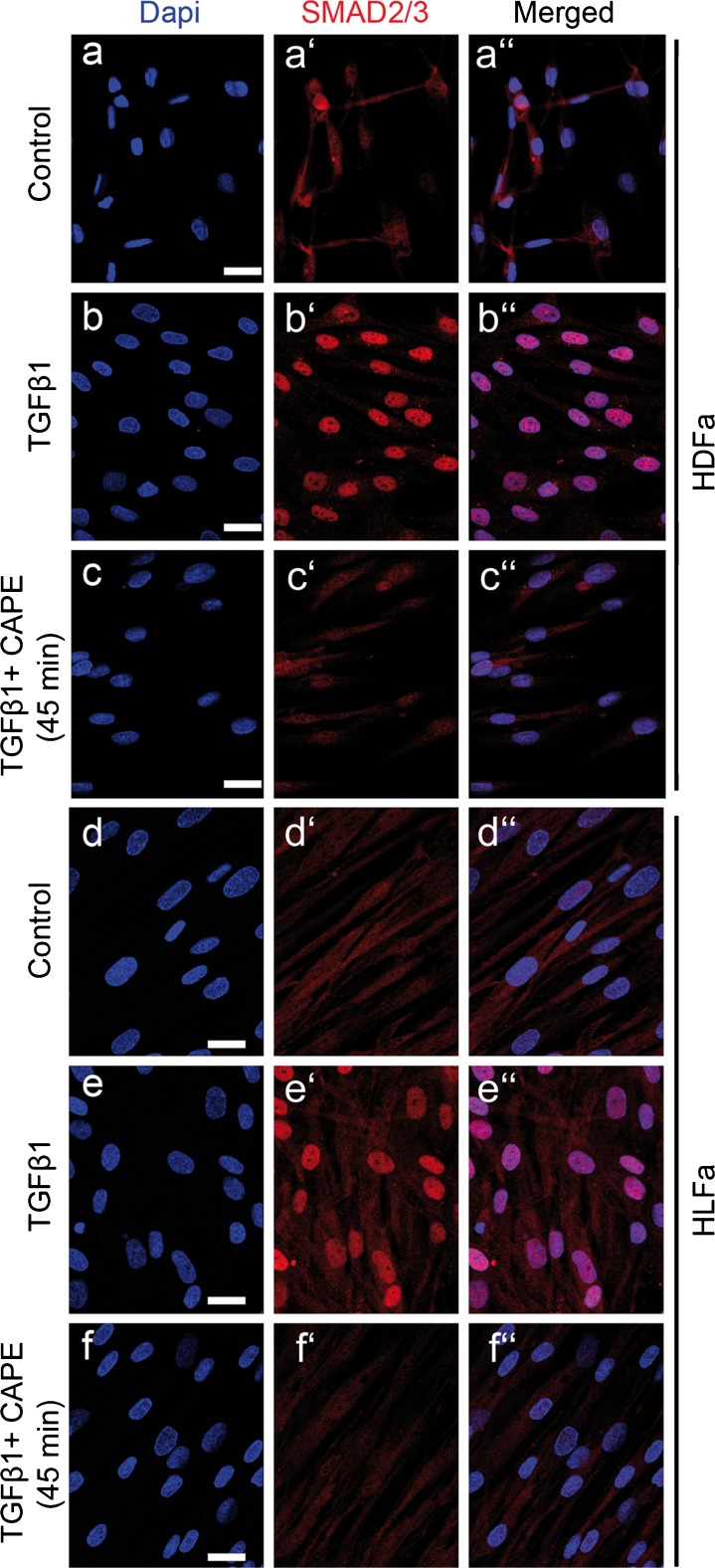
Effects of TGFβ1 and CAPE on nuclear localization of SMAD2/3. **a**–**c** HDFa and **d**–**f** HLFa were cultured for 45 min in the presence of TGFβ1 alone, or TGFβ1 in combination with CAPE (co-treatment). Fibroblasts cultured in the presence of TGFβ1 showed a strong nuclear localization of SMAD2/3 (**b**, **e**), whereas CAPE prevented the TGFβ1-induced nuclear translocation of SMAD2/3 (**c**, **f**). *Scale bar* 25 μm

Staining for SMAD2/3 showed a predominantly cytoplasmic localization in HDFa (Fig. 2a–c) and HLFa (Fig. 2d–f), a pattern that was dramatically shifted towards a nuclear localization when the cells were stimulated with TGFβ1 (Fig. 2b, e). CAPE clearly inhibited the nuclear translocation of SMAD2/3 (Fig. 2c, f). These observations are in line with that of Chuang et al. (2014, 2015).

### Effect of TGFβ1 and CAPE on ACTA2 (αSMA)

**Fig. 3:**
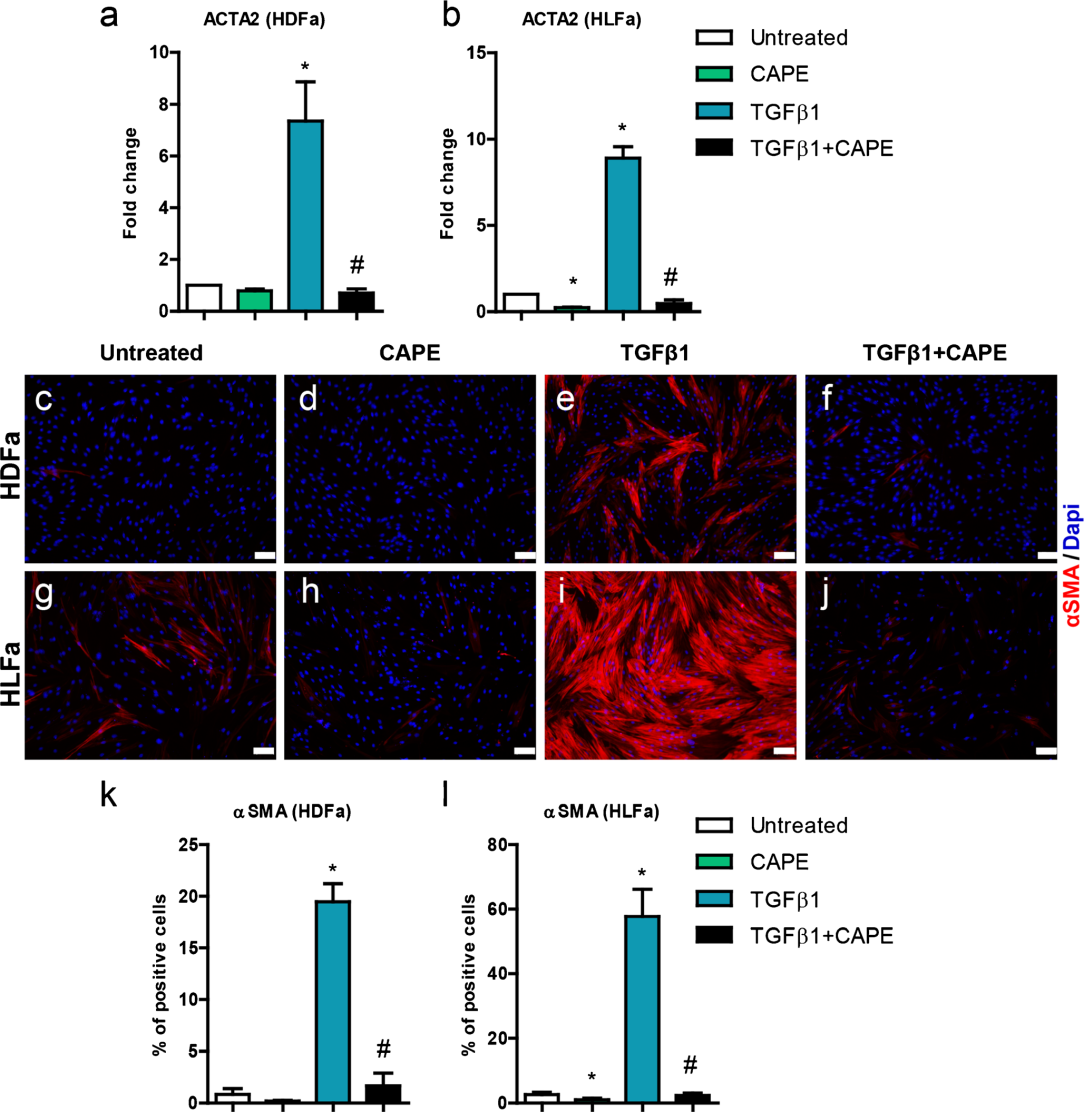
Effects of CAPE, TGFβ1 and TGFβ1 + CAPE on *ACTA2* mRNA levels and % αSMA-positive cells of HDFa and HLFa. Fibroblasts were cultured for 48 h in the presence of CAPE alone, TGFβ1 alone, or TGFβ1 in combination with CAPE (co-treatment). **a**, **b** mRNA levels of *ACTA2* relative to the reference gene *YWHAZ* and expressed as fold-change compared to untreated control (i.e. the baseline level as provided in Fig. 1). **c**–**j** Representative immunofluorescence stainings (*upper panel*) and **k**, **l** quantification of the % of cells (*lower panel*) positive for αSMA. * Statistically significant towards untreated control, and # statistically significant for cells co-treated with TGFβ1 + CAPE towards TGFβ1-treated cells. *Scale bar* 100 μm

**Fig. 4:**
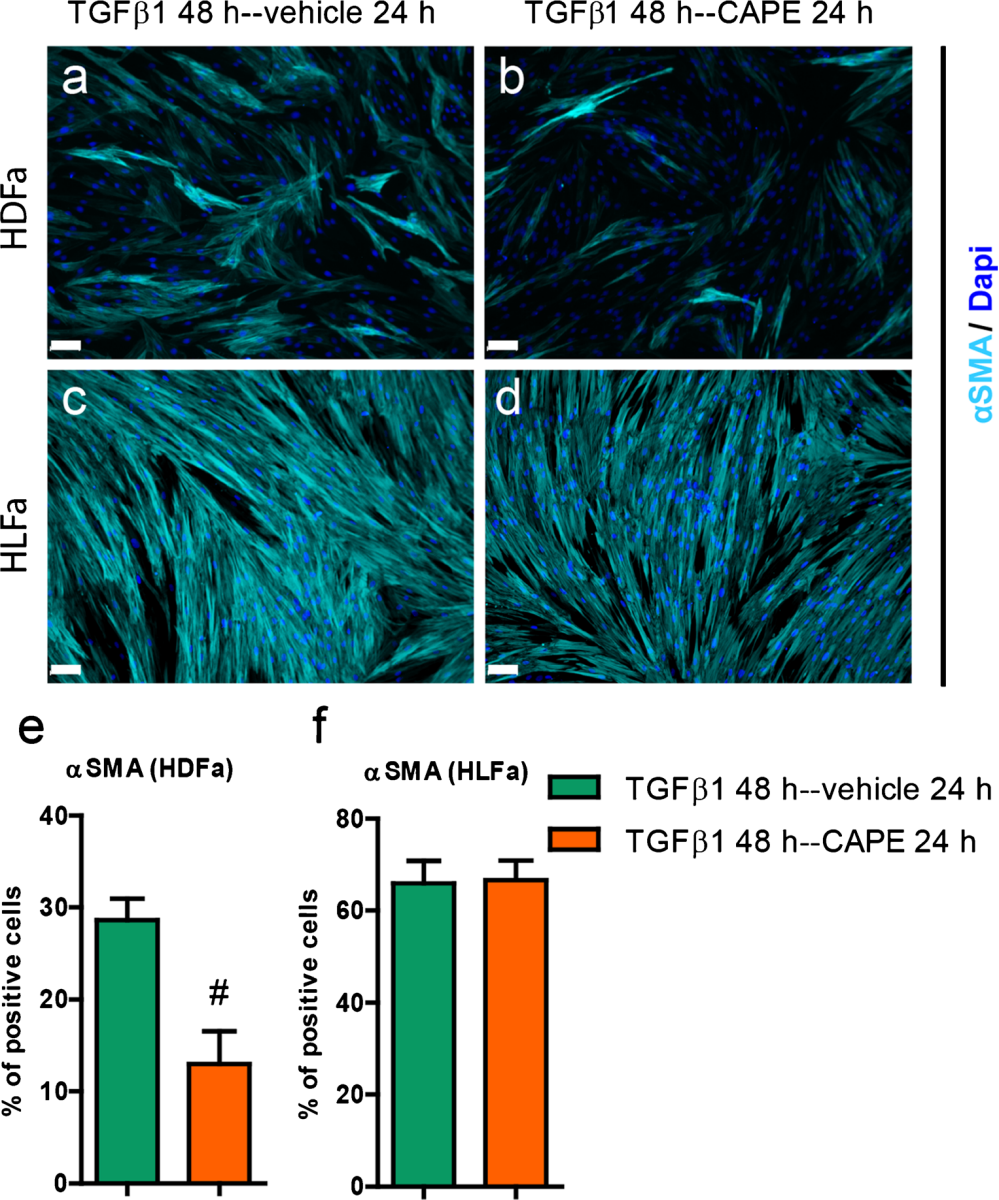
Effects of post-treatment of CAPE on cells treated with TGFβ1 regarding the % αSMA-positive cells of HDFa and HLFa. Fibroblasts were cultured for 48 h in the presence of TGFβ1, followed by a post-treatment with CAPE for 24 h. **a**–**d** Representative immunofluorescence stainings (*upper panel*) and **e**, **f** quantification of the % of cells (*lower panel*) positive for αSMA. # Statistically significant for cells post-treated with CAPE towards cells stimulated with TGFβ1 only. *Scale bar* 100 μm

In order to differentiate the fibroblasts into myofibroblasts, the cells were stimulated for 48 h with TGFβ1. Both HDFa and HLFa showed a major increase (>6-fold) in mRNA levels of *ACTA2* (encoding for the protein αSMA) (Fig. 3a, b), as was the case for the number of cells stained positive for αSMA (> 10-fold increase) (Fig. 3e, i, k, l). Interestingly, the addition of CAPE resulted in a suppression to baseline levels, both on an mRNA level (Fig. 3a, b) and on a protein level (Fig. 3f, j–l). Incubation of CAPE alone (thus without the addition of TGFβ1) also suppressed the mRNA and protein level in LFa, as these cells show, in contrast to HDFa, already at baseline in some αSMA positive cells (Fig. 3a–d, g, h, k, l). Thus, CAPE is able to inhibit the formation of TGFβ1-induced αSMA stress fiber formation. CAPE is, in addition, able to disrupt the already existing αSMA stress fibers seen in HLFa. However, a post-treatment with CAPE did not result in a decrease of the TGFβ1-induced αSMA stress fibers in HLFa, although a partial decrease was seen in HDFa under the same conditions (Fig. 4a–f).

### Effect of TGFβ1 and CAPE on TAGLN (SM22α)

**Fig. 5:**
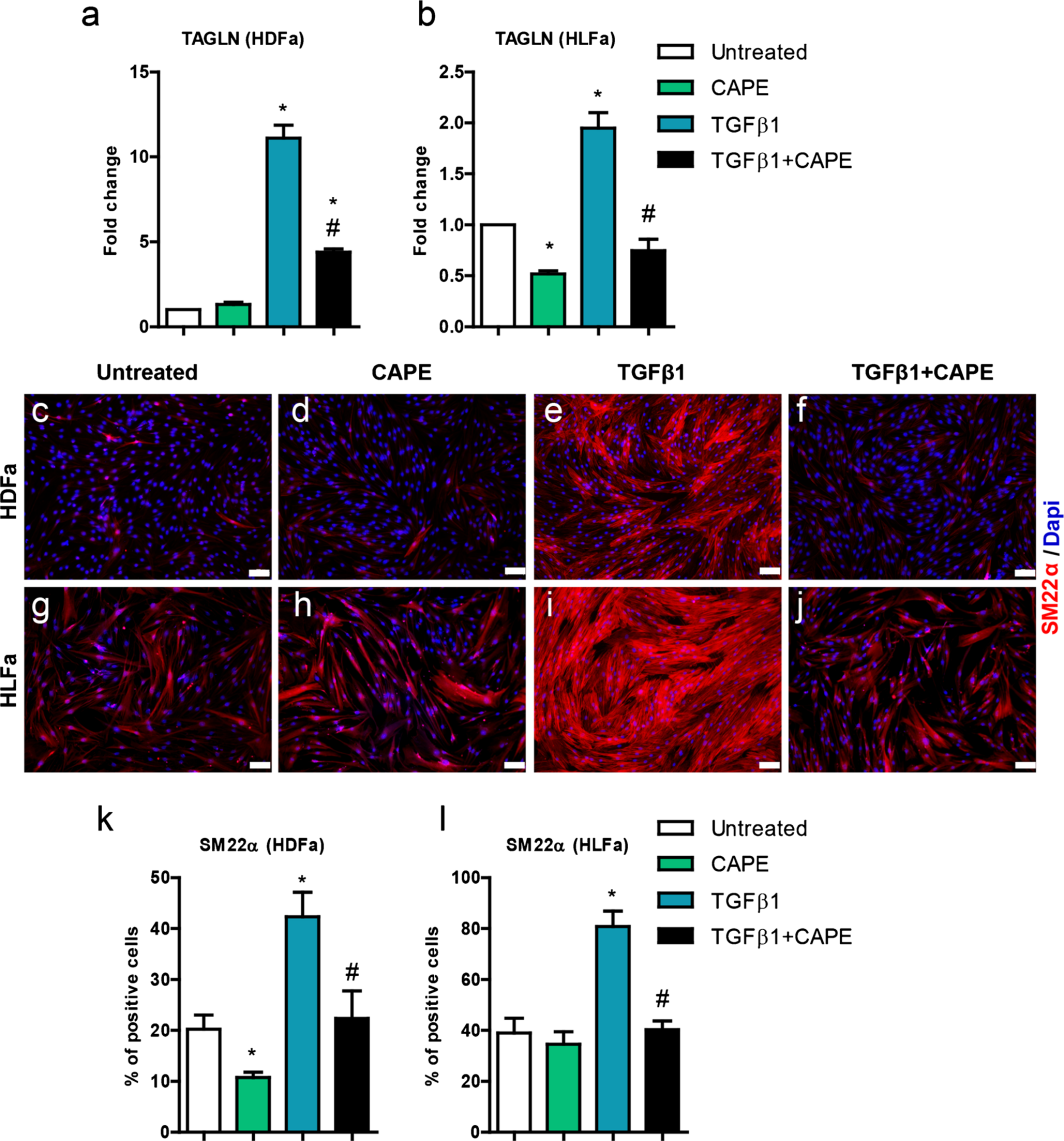
Effects of CAPE, TGFβ1 and TGFβ1 + CAPE on *TAGLN* mRNA levels and % SM22α-positive cells of HDFa and HLFa. Fibroblasts were cultured for 48 h in the presence of CAPE alone, TGFβ1 alone, or TGFβ1 in combination with CAPE (co-treatment). **a**, **b** mRNA levels of *TAGLN* relative to the reference gene *YWHAZ* and expressed as fold-change compared to untreated control (i.e. the baseline level as provided in Fig. 1). **c**–**j** Representative immunofluorescence stainings (*upper panel*) and **k**, **l** quantification of the % of cells (*lower panel*) positive for SM22α. * Statistically significant towards untreated control, and # statistically significant for cells co-treated with TGFβ1 + CAPE towards TGFβ1-treated cells. *Scale bar* 100 μm

Another cytoskeletal element, SM22α (encoded by *TAGLN*) showed similar results. mRNA was upregulated with TGFβ1 (HDFa: >10-fold; HLFa: 2-fold), an upregulation that was suppressed by CAPE, but reaches baseline levels only in HLFa (Fig. 5a, b). Stress fibers were upregulated with TGFβ1 as well, as indicated by a 2-fold increase in the % of cells that stain positive for SM22α. CAPE suppressed the number of positive cells to baseline levels, both for HDFa and HLFa (Fig. 5c–l). CAPE alone had an effect only on the number of positive cells that were present before TGFβ1 stimulation in HDFa (Fig. 5c, d, k).

### Effect of TGFβ1 and CAPE on collagen type I expression/formation

**Fig. 6:**
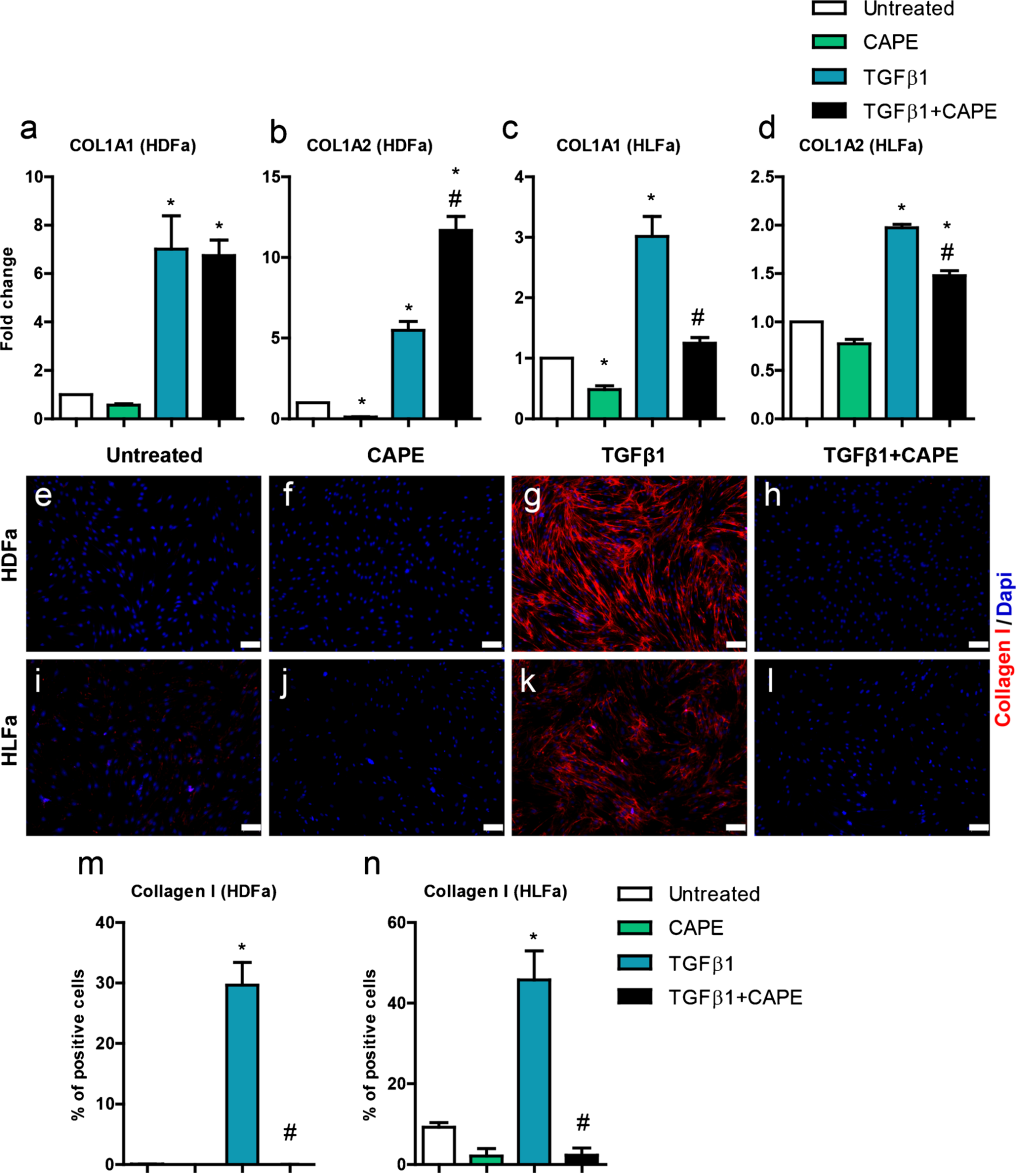
Effects of CAPE, TGFβ1 and TGFβ1 + CAPE on *COL1A1* and *COL1A2* mRNA levels and % collagen type I-positive cells of HDFa and HLFa. Fibroblasts were cultured for 48 h in the presence of CAPE alone, TGFβ1 alone, or TGFβ1 in combination with CAPE (co-treatment). **a**–**d** mRNA levels of *COL1A1* and *COL1A2* relative to the reference gene *YWHAZ* and expressed as fold-change compared to untreated control (i.e. the baseline level as provided in Fig. 1). **e**–**l** Representative immunofluorescence stainings (*upper panel*) and **m**, **n** quantification of the % of cells (*lower panel*) positive for collagen type I. * Statistically significant towards untreated control, and # statistically significant for cells co-treated with TGFβ1 + CAPE towards TGFβ1-treated cells. *Scale bar* 100 μm

**Fig. 7:**
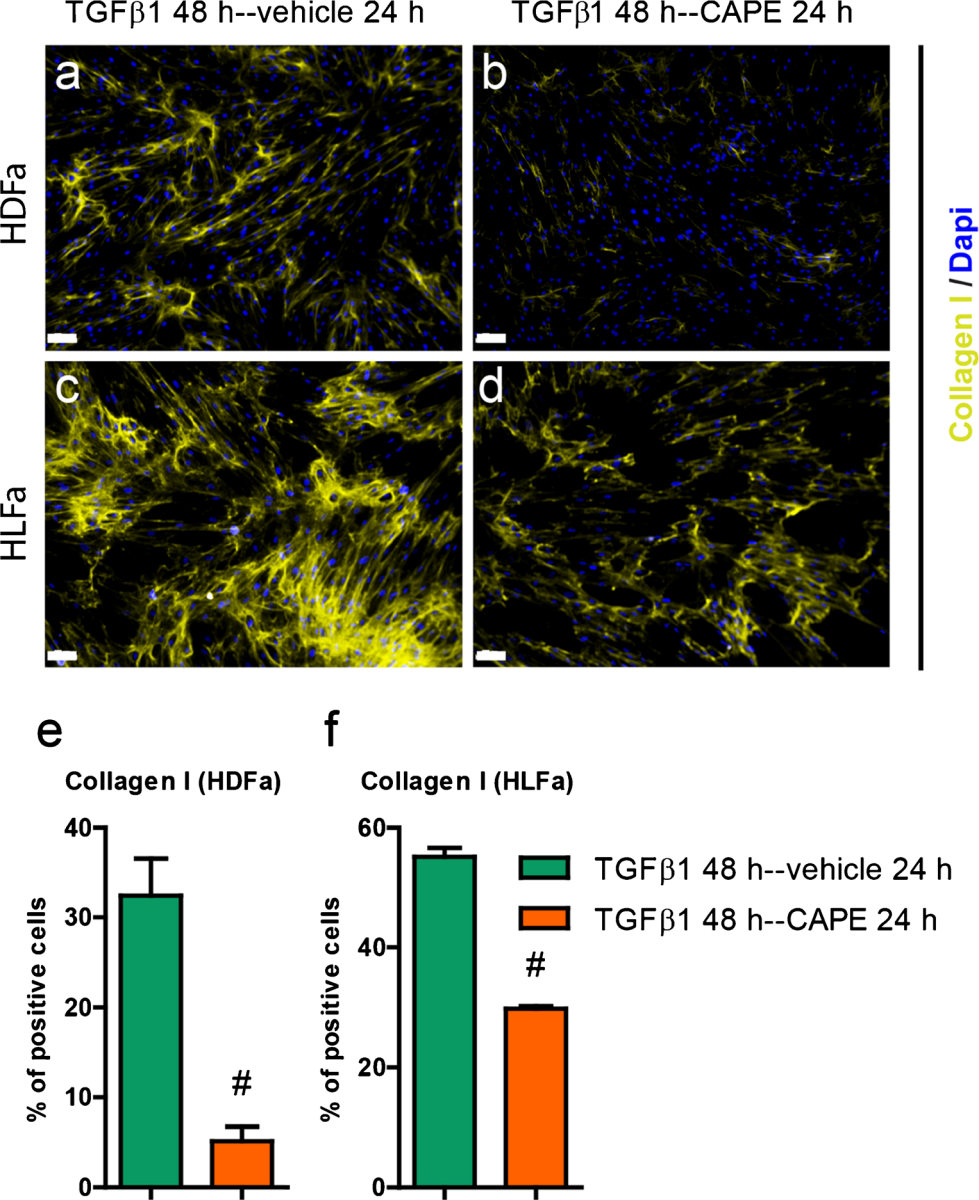
Effects of post-treatment of CAPE on cells treated with TGFβ1 regarding the % collagen type I-positive cells of HDFa and HLFa. Fibroblasts were cultured for 48 h in the presence of TGFβ1, followed by a post-treatment with CAPE for 24 h. **a**–**d** Representative immunofluorescence stainings (*upper panel*) and **e**, **f** quantification of the % of cells (*lower panel*) positive for collagen type I. # Statistically significant for cells post-treated with CAPE towards cells stimulated with TGFβ1 only. *Scale bar* 100 μm

We next investigated whether CAPE was able to inhibit collagen type I expression/formation. TGFβ1 increased the expression of *COL1A1* and *COL1A2* in HFDa and HLFa (7- and 5-fold and 2- and 3-fold, respectively) (Fig. 6a–d). CAPE did not inhibit the expression of *COL1A1* in HDFa (Fig. 6a), and increased the expression of *COL1A2* >2-fold (Fig. 6b). In contrast, CAPE inhibited the expression of *COL1A1* in HLFa to baseline levels (Fig. 6c), and slightly inhibited the expression of *COL2A1* (but not to baseline levels) (Fig. 6d). At the protein level, a different picture emerged: in both HDFa and HLFa, the % cells stained positive for collagen type I was dramatically reduced by CAPE. The few collagen-positive cells of HLFa were present before TGFβ1 stimulation also disappeared (Fig. 6e–n). Post-treatment with CAPE after TGFβ1 stimulation resulted in a major decrease in collagen-producing cells (HDFa: 5-fold decrease; HLFa: 2-fold decrease) (Fig. 7a–f).

### Effect of TGFβ1 and CAPE on PLOD2 (LH2)

**Fig. 8:**
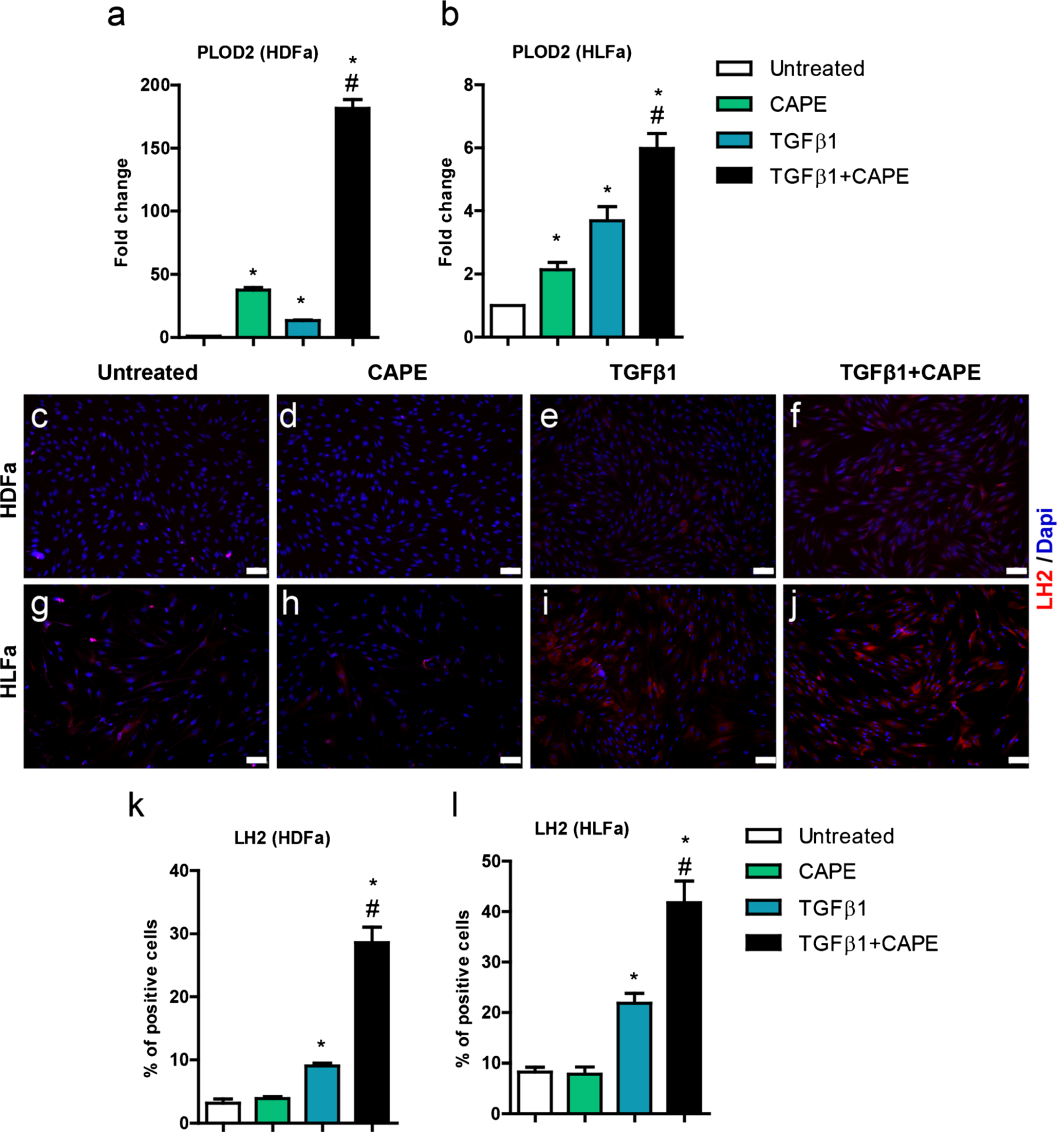
Effects of CAPE, TGFβ1 and TGFβ1 + CAPE on *PLOD2* mRNA levels and % LH2-positive cells of HDFa and HLFa. Fibroblasts were cultured for 48 h in the presence of CAPE alone, TGFβ1 alone, or TGFβ1 in combination with CAPE (co-treatment). **a**, **b** mRNA levels of *PLOD2* relative to the reference gene *YWHAZ* and expressed as fold-change compared to untreated control (i.e. the baseline level as provided in Fig. 1). **c**–**j** Representative immunofluorescence stainings (*upper panel*) and **k**, **l** quantification of the % of cells (*lower panel*) positive for LH2. * Statistically significant towards untreated control, and # statistically significant for cells co-treated with TGFβ1 + CAPE towards TGFβ1-treated cells. *Scale bar* 100 μm

In fibrotic collagen, increased amounts are seen of cross-links derived from hydroxylysine, a cross-link that is catalyzed LH2 (van der Slot 2004, 2005a; Yamauchi 2012). mRNA of *PLOD2*, encoding for LH2, was 15-fold and 4-fold upregulated after TGFβ1 stimulation in HDFa and HLFa, respectively (Fig. 8a, b). Both cell types reacted quite differently towards CAPE with respect to *PLOD2*. In HDFa, *PLOD2* mRNA levels were more than 10-fold upregulated compared to the mRNA levels seen with TGFβ1 alone (Fig. 8a); in HLFa, this increase was <2-fold (Fig. 8b). Cells that were not stimulated with TGFβ1 also showed an upregulation of *PLOD2* when incubated with CAPE: the induction was about 40-fold in HDFa and 2-fold in HLFa (Fig. 8a, b). The increase of mRNA levels of *PLOD2* without the presence of TGFβ1 did not result in an increase of the % cells that stained positive for LH2. The % LH2-positive cells increased when stimulated with TGFβ1, and increased an additional 1.5- to 2-fold in the presence of CAPE (Fig. 8c–l).

### Effect of TGFβ1 and CAPE on fibronectin (FN1) and tenascin C (TNC) expression

**Fig. 9:**
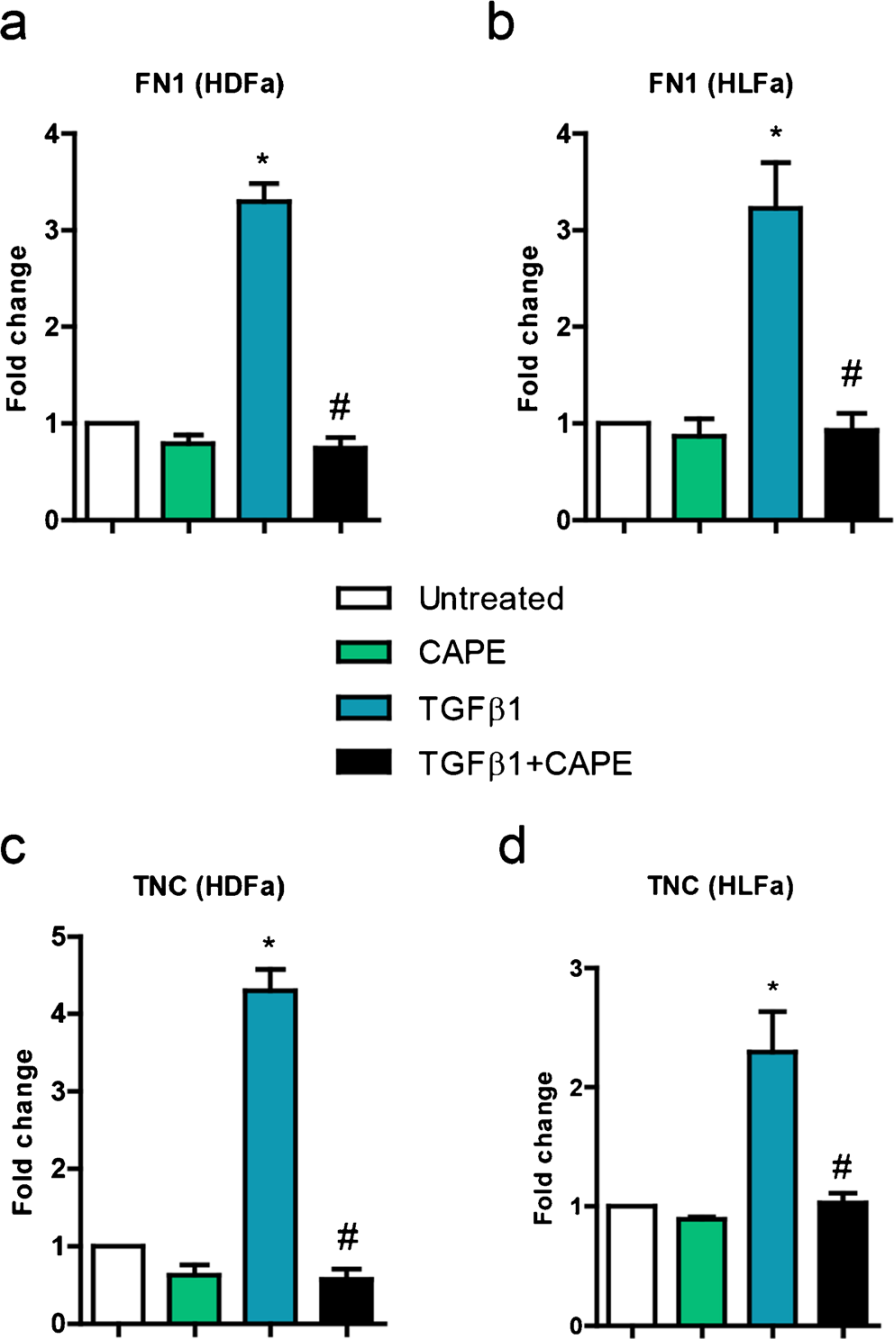
Effects of TGFβ1 and CAPE on fibronectin (*FN1*) and tenascin C (*TNC*) gene expression. **a**–**d** Fibroblasts were cultured for 48 h in the presence of CAPE alone, TGFβ1 alone, or TGFβ1 in combination with CAPE (co-treatment) on mRNA levels of *FN1* and *TNC* relative to the reference gene *YWHAZ* and expressed as fold-change compared to untreated control. * Statistically significant towards untreated control, # statistically significant for cells co-treated with TGFβ1 + CAPE towards TGFβ1-treated cells

Apart from collagen type I, we also tested the effect of CAPE on the expression and synthesis of the extracellular matrix proteins FN1 and TNC. TGFβ1 increased the expression of *FN1* and *TNC* in HFDa and HLFa (Fig. 9a–d), an upregulation that was suppressed to baseline levels by CAPE (Fig. 9a–d). The same was seen with respect to the % cells that stained positive for FN1 and TNC (data not shown).

### Effect of TGFβ1 on GLI, GLI2 and SNAIL1

**Fig. 10:**
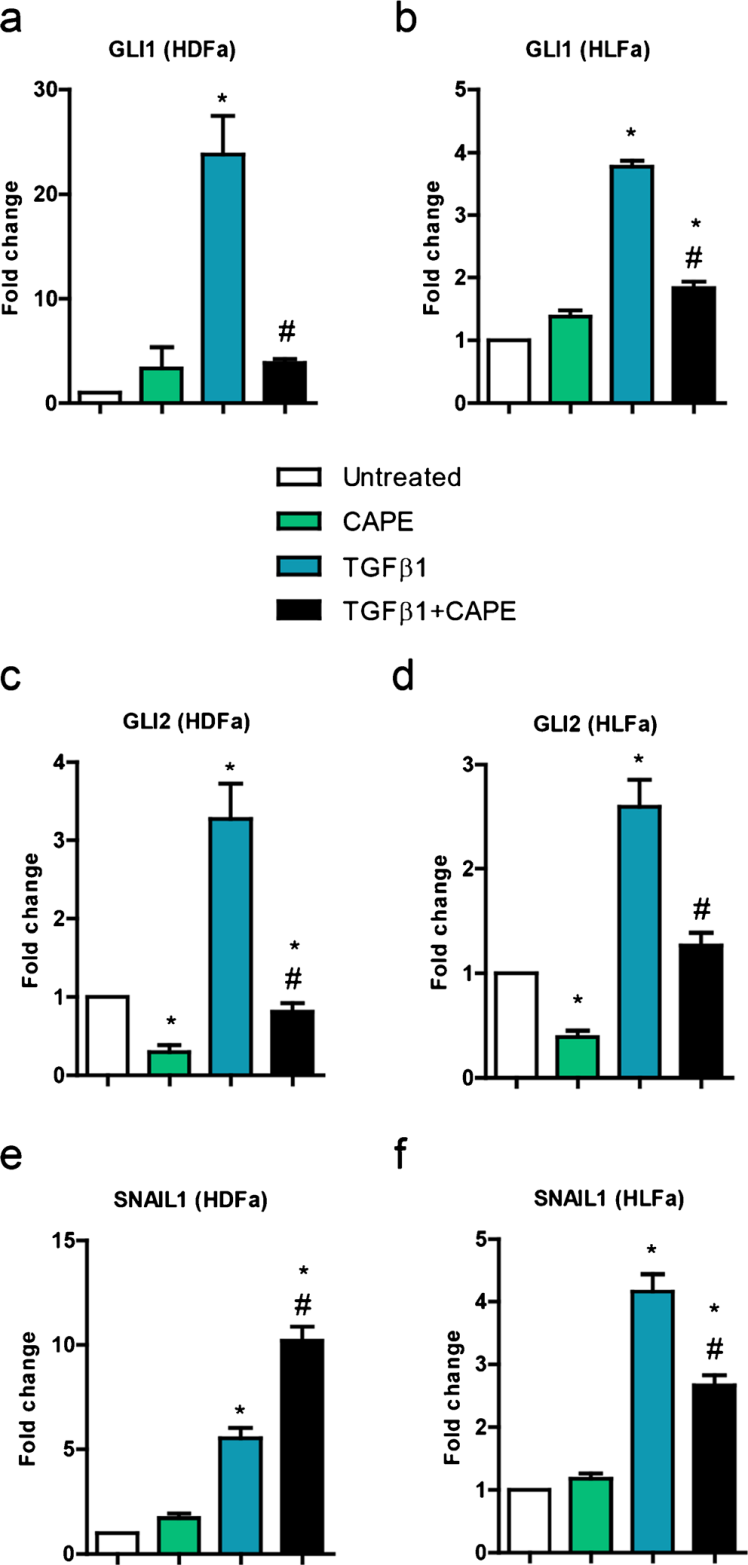
Effects of CAPE, TGFβ1 and TGFβ1 + CAPE on *GLI1, GLI2* and *SNAIL1* mRNA levels of HDFa and HLFa. **a**–**f** Fibroblasts were cultured for 48 h in the presence of CAPE alone, TGFβ1 alone, or TGFβ1 in combination with CAPE (co-treatment) on mRNA levels of *GLI1, GLI2* and *SNAIL1* relative to the reference gene *YWHAZ* and expressed as fold-change compared to untreated control (i.e. the baseline level as provided in Fig. 1). * Statistically significant towards untreated control, # statistically significant for cells co-treated with TGFβ1 + CAPE towards TGFβ1-treated cells

The presence of the transcription factors GLI1 and GLI2 are required for myofibroblast formation. As expected, expression of *GLI1* and *GLI2* was upregulated in the presence of TGFβ1: >20-fold and 3-fold in HDFa, respectively (Fig. 10a, c), and about 4-fold and 2.5-fold in HLFa, respectively (Fig. 10b, d). The presence of CAPE during TGFβ1 stimulation had a major inhibitory effect on TGFβ1-induced upregulation of *GLI1* and *GLI2* (Fig. 10a–d). The transcription factor SNAIL1 was also upregulated in the presence of TGFβ1: a 6-fold and 4-fold induction was seen for HDFa and HLFa, respectively. Remarkably, the presence of CAPE during TGFβ1 stimulation resulted in an even higher upregulation of *SNAIL1* in HDFa, whereas a downregulation was seen in HLFa (Fig. 10e, f).

## Discussion

A generally accepted model to mimic fibrotic events in vitro is the stimulation of fibroblasts with TGFβ1, a cytokine that shows strong pro-fibrotic properties. TGFβ1 stimulates the synthesis of collagen, and also differentiates fibroblasts into myofibroblasts. Although CAPE has been found to inhibit fibrosis in vivo in rodents, very little is known about whether CAPE has direct anti-fibrotic properties on fibroblasts. Here, we show that human dermal and lung fibroblasts treated with TGFβ1 result in high numbers of myofibroblasts (i.e. cells that show αSMA) and considerable protein production of collagen type I (as revealed by a major increase in the number of cells stained positive for collagen), and that co-incubation with CAPE entirely blocks both fibrotic hallmarks. Thus, CAPE has strong anti-fibrotic properties towards fibroblasts. This statement can even be made despite the high increase in *PLOD2* mRNA levels (and LH2 protein synthesis). LH2 is a major pro-fibrotic enzyme as it plays a role in the formation of hydroxylysine-derived collagen cross-links (van der Slot et al. 2003, 2004, 2005a). Collagens containing this type of cross-links are especially difficult to degrade (van der Slot et al. 2005b; Brinckmann et al. 2005). However, in the presence of CAPE, hardly any collagen is produced.

We next investigated whether CAPE is able to reverse myofibroblasts into fibroblasts, and if this is reflected in decreased expression levels of collagen. Remarkable differences were seen between HDFa and HLFa: post-treatment with CAPE showed a 2-fold decrease in αSMA-positive cells in HDFa, whereas no decrease was seen in αSMA-positive cells in HLFa. However, in both cases, a decrease is seen in the % collagen-positive cells: a 5-fold decrease was observed for HDFa, and a 2-fold decrease for HLFa. It is of importance to note that CAPE thus has the capacity to partially reverse myofibroblasts into fibroblasts (in the case of HDFa) and/or partially inhibit collagen formation by myofibroblasts (in the case of HLFa). Reversal of myofibroblasts to fibroblasts has so far only been described for a few compounds (Yang et al. 2014). The data of HLFa show that a decrease in collagen formation can occur despite unchanged levels of αSMA stress fibers. Indeed, the relationship between αSMA and collagen is not 1:1, as it has been shown that αSMA^-/-^ myofibroblasts are still able to produce collagen (Takeji et al. 2006).

Since the presence of GLI1 and GLI2 is necessary for myofibroblast formation, and CAPE is able to inhibit myofibroblast formation as induced by TGFβ1, we wondered vwhether the co-incubation of TGFβ1 with CAPE has an effect on *GLI1* and *GLI2* mRNA levels. Induction of GLI2 by TGFβ requires a nuclear presence of SMAD3 (Dennler et al. 2007, 2009; Javelaud et al. 2011). Furthermore, *GLI1* expression as conducted by TGFβ requires *GLI2* (Dennler et al. 2007). The decrease of *GLI2* as seen in the presence of CAPE is therefore likely to be related to the low levels of SMAD3 in the nucleus, as observed by confocal laser scanning microscopy. In addition, it is reasonable to assume that the decrease of *GLI1* in the presence of CAPE is a consequence of diminished levels of *GLI2*, as GLI1 acts downstream of GLI2.

The binding of GLI1 and GLI2 to their DNA targets can be blocked by the compound GANT61 (Lauth et al. 2007). Fibroblasts stimulated with TGFβ1 in the presence of GANT61 did not show myofibroblast formation, collagen formation was not upregulated, and αSMA stress fiber formation was not observed (Cigna et al. 2012). In our studies with CAPE, we observed the same, and it coincided with the suppression of *GLI1* and *GLI2* expression to baseline levels. It is therefore tempting to speculate that the observations with CAPE regarding collagen type I and αSMA can at least be partially ascribed to the ability of CAPE to suppress *GLI1* and *GLI2* expression. On the other hand, post-treatment studies with GANT61 on TGFβ1-stimulated fibroblasts showed a complete reversal of myofibroblasts into fibroblasts, as revealed by an inhibition of collagen type I formation and the absence of αSMA stress fibers (although protein levels of αSMA were hardly affected) (Cigna et al. 2012). In contrast, post-treatment with CAPE did not completely reverse collagen production (both for HDFa and HLFa) and stress fiber formation (for HDFa), or did not reverse stress fiber formation at all (for HLFa). Thus, the post-treatment as seen with CAPE does not follow the same pattern of GANT61 post-treatment, which can be explained by assuming that the remaining GLI1 and GLI2 levels are still (partially) capable of binding to their DNA targets.

Our data show that mRNA data of *COL1A1* and *COL1A2* cannot be used to predict collagen formation. In HDFa, mRNA levels of *COL1A1* and *COL1A2* remained the same and increased, respectively, when CAPE was added to the TGFβ1, but the % cells stained positive for collagen decreased dramatically. The fold-decrease of *COL1A1* and *COL2A1* in HLFa was also not in line with the fold decrease in the % collagen-positive cells. Studies regarding the anti-fibrotic properties of compounds should therefore always take protein data as leading, not mRNA levels, at least with respect to collagen. No conflicting data were observed between mRNA levels and protein data of fibronectin and tenascin C.

Finally, there are major differences in the response of HDFa and HLFa towards CAPE. For example, when CAPE is added to TGFβ1, mRNA levels of *COL1A2* are even further upregulated, whereas a decrease is seen in HLFa. This was also the case for *SNAIL1*. In addition, the magnitude of response can differ markedly. mRNA levels of *PLOD2* were highly upregulated in HDFa when CAPE was added to the culture medium, either alone or in combination with TGFβ1, whereas a modest upregulation of *PLOD2* was seen in HLFa. It is well known that there is a high heterogeneity among fibroblast strains, not only between tissues but also within a single tissue (Chang et al. 2002; Sorrell and Caplan 2009). It remains to be investigated whether our data reflect tissue-specific or strain-specific differences; in either case, CAPE was able to inhibit myofibroblast formation.

In conclusion, we show that CAPE has direct anti-fibrotic effects on fibroblasts: it is able to fully counteract the TGFβ1-induced myofibroblast formation and concomitant collagen formation, and is even able to partially reverse myofibroblasts into fibroblasts and/or partially reverse collagen formation.
